# Investigation of multiple commercial electrocatalysts and electrocatalyst degradation for fuel cells in real vehicles

**DOI:** 10.1039/d2ra05682h

**Published:** 2022-11-11

**Authors:** Wenhui He, Yanjuan Xiang, Mudi Xin, Limei Qiu, Wenyan Dong, Wenhui Zhao, Yuxia Diao, Aiguo Zheng, Guangtong Xu

**Affiliations:** SINOPEC Research Institute of Petroleum Processing Co., Ltd. Beijing 100083 China xugt.ripp@sinopec.com +86 10 82368613

## Abstract

Proton exchange membrane fuel cells (PEMFCs) are regarded as one of the promising new carbon mitigation strategies to realize carbon neutrality. However, efficient and robust electrocatalysts are vital for the commercialization of PEMFCs. Herein, three commercial Pt/C electrocatalysts were investigated including a carbon support and Pt nanoparticles (NPs) to identify their merits and disadvantages, which will help end users quickly select catalysts with excellent performances among the many brands of domestic and foreign catalysts to further better study and better utilize them. Subsequently, they were optimized for real automotive application for about 1800 h, and then the variations in the electrocatalysts on the MEA were analysed by transmission electron microscopy (TEM) and X-ray photoelectron spectroscopy (XPS). The mean Pt particle size of the catalysts after operating for 1800 h (cathode, 9.9 ± 3.2 nm) was nearly 4-fold that before use (2.5 ± 0.6 nm), greatly reducing the exposure of metal sites, which was due to the violent three-phase interfacial reaction (ORR) occurring at the cathode side. Also, this assertion was supported by the negative shift in the Pt 4f peaks in the XPS spectra. Moreover, to determine the coalescent evolution of the Pt particles, an *in situ* TEM experiment was performed. This allowed us to perform fundamental Pt NP degradation studies on the carbon support, which can result in an improvement in the sustainability of catalysis.

## Abstract 2

The coalescence of Pt nanoparticles during operation in a real vehicle is considered to be the main reason to weaken the ORR. The trajectories of oriented attachment were disclosed by observing the coalescence events of Pt NPs using *in situ* TEM.

## Graphical abstract

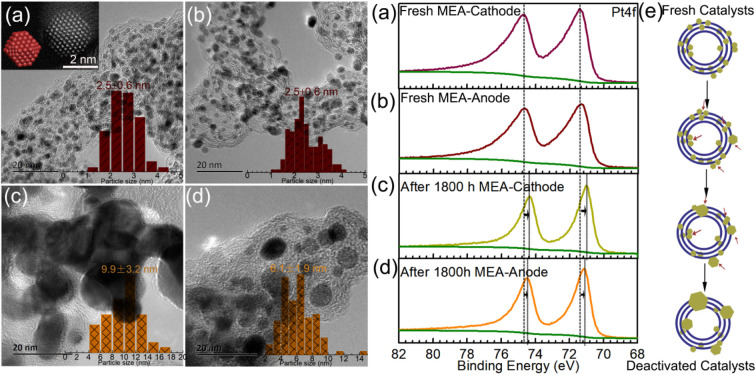

## 1 Introduction

Proton exchange membrane fuel cells (PEMFCs) are attracting continuing interest due to their advantages, such as high energy conversion efficiency, high power density, short start-up, and absence of gas pollution, which are a potential carbon mitigation strategy to realize carbon neutrality.^1–3^ With the rapid development of PEMFC technology, many automotive manufacturers have brought PEMFC electric vehicles to the market. However, to date, fuel-cell systems are relatively very expensive compared with the traditional internal combustion engines. The current high cost of fuel cell systems arises from their electrocatalyst component given that it is one of the core materials in PEMFCs, accounting for more than 50% of the stack cost, which is one of roadblocks in the commercialization of PEMFCs.^4^ In numerous studies, electrocatalysts, including Pt-alloy catalysts with transition metals and non-noble metal catalysts, have been investigated to reduce the Pt amount, and thus further improve the cost effectiveness.^5–8^ In the study of low-cost electrocatalysts, commercial carbon-supported platinum (Pt/C) catalysts are often used as the object of comparison, but the performance of the commercial catalysts is not consistent.^2,9–13^ Furthermore, although numerous low-cost electrocatalysts have been extensively investigated,^14^ Pt/C catalysts remain the most active electrocatalysts for PEMFC electric vehicles to date due to the drawbacks of low-cost catalysts, which hinder their practical use. There are several brands of Pt/C catalysts both domestically and overseas, such as Shanghai Hesen Electronics Co. Ltd., Johnson Matthey, and Tanaka Kikinzoku Kogyo K.K. Therefore, it is imperative to assess the cost-effectiveness of the commercial Pt/C catalysts in research and actual application. However, a complete description of multiple commercial Pt/C catalysts is relatively scarce to date.

Also, catalyst degradation is a serious problem during the practical application of PEMFCs. Previous studies have shown the degradation mechanisms of Pt/C catalysts, which involve the following three aspects: (1) electrochemical Ostwald ripening,^15–17^ (2) coalescence of Pt NPs *via* aggregation or migration on the carbon support,^4,18^ and (3) Pt NP agglomeration triggered by the corrosion of the carbon support.^4,19^ Current research and development efforts are devoted to elucidating and quantifying the above-mentioned proposed mechanisms by various approaches. In general, in terms of Pt NP growth, the pathways can be classified into two categories. One is the Pt NP transport on the carbon support surface through migration, followed by coalescence, and the other is the evaporation of Pt atoms from one particle, and then their condensation on another one in a process. Sun *et al.*^20^ reported the coalescence behavior of Pt NPs and illustrated the strong anchoring effect of TaO_*x*_ towards Pt NPs, which hinders the migration, coalescence and detachment of Pt NPs from the support. However, it lacked intuitive observation, which hampers in depth insight into the degradation mechanisms. Analytical *in situ* transmission electron microscopy (TEM) offers a powerful tool to directly visualize the microstructural variation at the nanometer scale and atomic scale during reactions,^21–24^ thus making it feasible to investigate the dynamic microstructural variation in Pt/C catalysts. Wu *et al.*^25^ performed a series of *in situ* liquid cell TEM experiments to quantitatively analyze the dissolution kinetics of Pt NPs in a mixed solution of HAuCl_4_ and KCl, where the etching rate of the Pt NP corners is much larger than that of the edges and the terraces. Yao *et al.*^26^ used *in situ* TEM to investigate the coalescence mechanism of carbon-supported Pt-alloy catalysts (such as PtFe_3_, PtCo_3_, and PtNi_3_) as a function of temperature, highlighting the different coalescence behaviors of different Pt-alloy catalysts. Although *in situ* TEM characterization is used in some reports on Pt-based electrocatalysts, the structural evolution of Pt NPs in commercial Pt/C catalysts during their operation is still insufficient based on current research, and thus further research is needed.

In this work, we present a complete description of three commercial Pt/C catalysts, which can identify their merits and disadvantages to help screen efficient and robust electrocatalysts among catalyst manufacturers that meet long-term performance degradation requirements. Subsequently, the optimized Pt/C catalysts were assembled into *s* membrane electrode assembly (MEA), and then their real fuel cell vehicle application was realized. After operating for 1800 h, the performance of the Pt/C catalysts degraded. We identified the variations in the catalysts by comparing them with the corresponding fresh catalyst. To further understand the degradation mechanism, *in situ* structural observation of the Pt NPs was performed by TEM combined with a temperature-driven process. The results pave the way to reveal an atomistic view of the transformation processes of the Pt component during the application of catalysts and can help formulate design rules for developing advanced carbon supports and metal components.

## 2 Experimental

### 2.1 Catalyst characterization

Commercial electrocatalysts (Pt/C, 54 wt% Cat.A, 47 wt% Cat.B, and 40 wt% Cat.C) were purchased from different catalyst manufacturers. All electrocatalysts were investigated as received without any treatments. Comparative studies on the carbon supports and active metals for the three commercial Pt/C catalysts were carried out using multiple methods. The specific surface area and pore size distribution of the electrocatalysts were obtained from nitrogen adsorption–desorption curves according to the Brunauer–Emmett–Teller (BET) and *t*-plot method, respectively (Micromeritics ASAP 2002). Raman spectra were collected at 532 nm laser excitation using a Horiba-Jobin-Yvon Raman system equipped with a microscope. The phase structure was identified using a Bruker D5005 X'Pert X-ray diffraction (XRD) diffractometer equipped with Cu Kα radiation (*λ* = 1.5406 Å). X-ray photoelectron spectroscopy (XPS) was performed using a Thermo Fisher ESCALab 250xi^+^ spectrometer with an Al Kα source (C 1s peak 284.5 eV as reference for calibration). The morphology of the catalysts was characterized using a transmission electron microscope (TEM, FEI Tennai F20). Given that aberration-corrected scanning transmission electron microscopy (Cs-STEM) can provide atomic-scale features, Cs-STEM high-angle annular dark-field (Cs-STEM-HAADF, JEM-ARM200F) was used to obtain the detailed structure of the active components. Combined with an *in situ* holder (DENSsolutions), *in situ* TEM was realized to monitor the structural evolution of the Pt NPs driven by temperature.

### 2.2 Electrochemical measurements

Electrochemical measurements were conducted on a CHI760E electrochemical station using a rotating disk electrode (RDE) (diameter: 5 mm; area: 0.19635 cm^2^) in a typical three-electrode system.^27^ The working electrode was prepared as follows: 2 mg catalyst was dispersed ultrasonically in 1 mL ethanol and 20 μL 5 wt% Nafion solution. Then 10 μL suspension was pipetted on *s* glass carbon electrode as working electrode and dried in air. The cycle voltammetry (CV) curves were recorded in N_2_-saturated 0.1 mol L^−1^ HClO_4_ at a scan rate of 50 mV s^−1^. Linear sweep voltammetry (LSV, ORR polarization) curves were measured in O_2_-purged 0.1 mol L^−1^ HClO_4_ electrolyte at a sweep rate of 50 mV s^−1^ at 1600 rpm.

### 2.3 Particulars of real fuel cell vehicle application

The optimized Cat.C was translated to a real-world MEA to test the real vehicle performance by Hydrogen Energy Co., Ltd. The electric stack started to run on the road on December 1, 2019. After the failure alarm of low overall performance occurred on December 8, 2020, the electric stack was removed from the vehicle.

On the monitoring platform, the average monthly travel was 4596 km, and the operating mileage of the electric stack was 4921 km, which is roughly estimated to have been running on the vehicle for 10 months. The monitoring platform showed that it ran for 5–6 h daily, with the highest running speed of 50–55 km h^−1^, and the average speed was 18–21 km h^−1^. The average daily mileage was 153 km, and it started and stopped about 8 times a day. Most of the time, the daily start time was about 6 am and the stop time was about 6 pm. In each run period, the duration time was 25–60 min and the down time was 25–50 min.

## 3 Results and discussion

### 3.1 Catalyst characterization

**Fig. 1:**
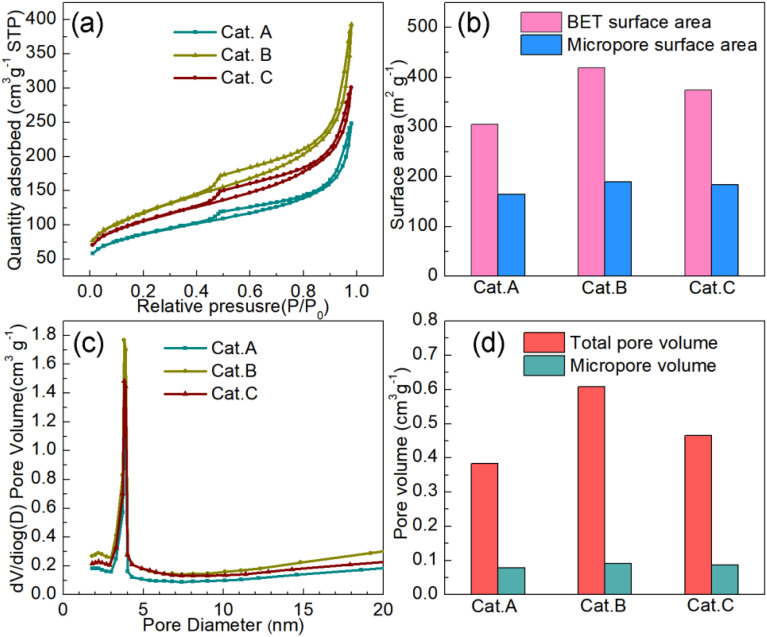
(a) N_2_ adsorption–desorption isotherms of three commercial catalysts. Summary of their specific surface area (b), pore size distribution (c) and pore volume (d).

It is obvious that the carbon support of PEMFC catalysts has been developed quite well, which is essential for the performance of PEMFCs. The requirements for the carbon support include high specific surface area, excellent conductivity, and adequate corrosion resistance.^28^ The rich mesopores of the carbon support can provide a strong dispersion force for Pt NPs, and thus a robust catalyst should have a considerable amount of mesopores. To understand the specific surface areas and pore structures of the three commercial catalysts, N_2_ adsorption–desorption tests were carried out, as depicted in Fig. 1a, which show that they all have obvious H_4_-type hysteresis loops. This profile indicates the visible existence of a hierarchical micropore and mesopore structure. The specific surface areas of Cat.A, Cat.B and Cat.C are 305 m^2^ g^−1^, 419 m^2^ g^−1^, and 374 m^2^ g^−1^, respectively, which illustrate that Cat.B and Cat.C have abundant pore structures (Fig. 1b). Moreover, the proportions of the mesopore surface area and the mesopores volume further indicate that Cat.B and Cat.C can provide more oxygen to Pt NPs to reduce the mass transfer resistance because mesopores act as a gas diffusion channel (Fig. 1b–d).^10,29^ Also, according to the literature, Pt NPs mainly exist in mesopores as the oxygen reduction reaction (ORR) catalytic sites.^29,30^ Hence, with regard to the carbon support only, the support of Cat.B presents an impressive pore structure for Pt/C catalysts, closely followed by the support of Cat.C.

**Fig. 2:**
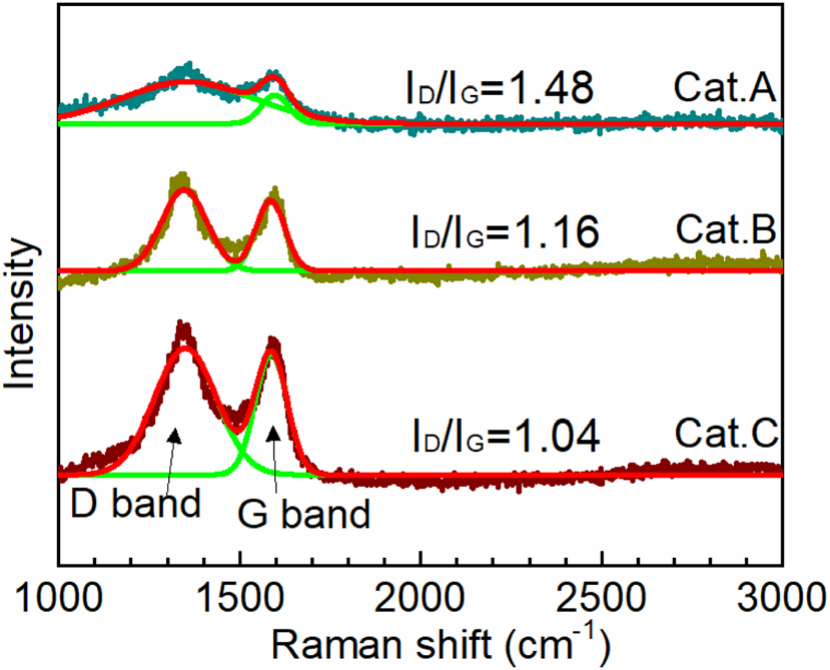
Raman spectra recorded for Cat.A, Cat.B and Cat.C.

To provide insight toward understanding the structure of the carbon support of the three catalysts, their Raman spectra were recorded. It should be noted that for carbon materials, the peak at 1350 cm^−1^ (D-band) reflects the disorder and defects, while that at 1580 cm^−1^ (G-bond) is related to the sp^2^-hybridized carbon.^31,32^ Their intensity ratio (*I*_D_/*I*_G_) reflects the degree of long-term order in the graphite lattice, which is defined as the graphitization degree. The lower the *I*_D_/*I*_G_ value, the better the graphitization degree. Fig. 2 clearly shows that the graphitization degree increases in the order of *I*_D_/*I*_G(Cat.A)_ > *I*_D_/*I*_G(Cat.B)_ > *I*_D_/*I*_G(Cat.C)_, highlighting that the support of Cat.C has an optional carbon structure. This result further indicates that the support of Cat.C has good conductivity and corrosion resistance, which effectively boost the electron transport and restrain the detachment, agglomeration, and growth of Pt NPs. In summary, considering the porosity and carbon structure, the support of Cat.C has the best comprehensive performance for electrocatalysts.

**Fig. 3:**
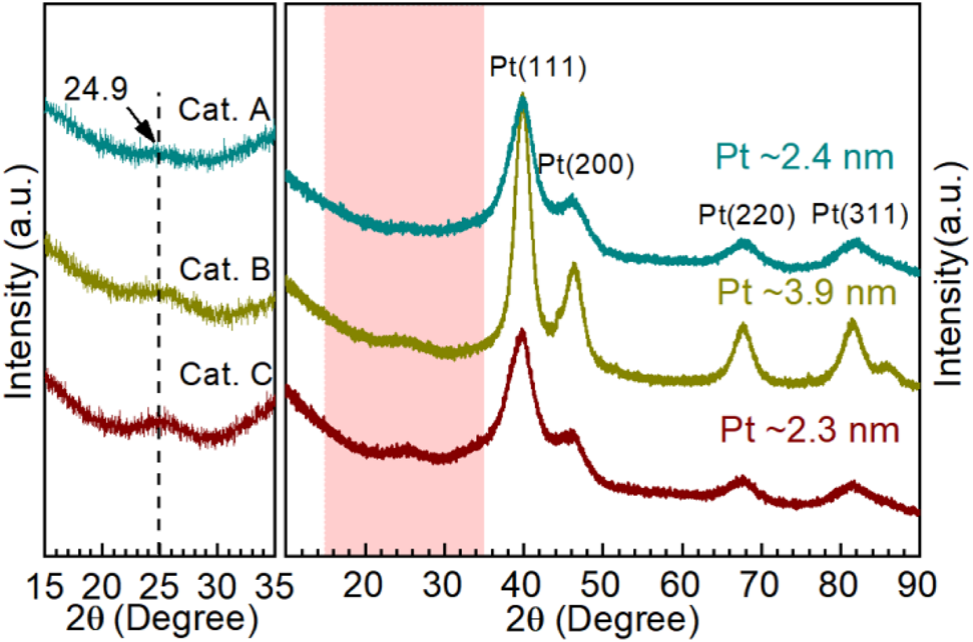
XRD patterns of Cat.A, Cat.B and Cat.C, where the light pink area is enlarged to clearly show the structural information of the carbon supports and the average grain size (Cat.A: ∼2.4 nm, Cat.B: ∼3.9 nm, and Cat.C: ∼2.3 nm) was calculated according to the Scherrer formula.

Moreover, X-ray diffraction (XRD) was performed to investigate the catalyst phase composition, as illustrated in Fig. 3. Firstly, the enlarged area shows the information on the carbon support. The support of Cat.C has an obvious graphitization C (002) peak, which is consistent with the above-mentioned Raman result. Furthermore, the typical face-centered cubic structured Pt for Cat.A, Cat.B and Cat.C could be identified to the corresponding Miller indices. It should be noted that the peak of Pt in Cat.B is narrower and more intense than that of the other two catalysts, which manifests the better crystallinity and larger average grain size of the Pt NPs in Cat.B. The theoretical Pt loading of the three catalysts is 54 wt%, 47 wt%, and 40 wt%, respectively, which indicates the good crystallinity of Pt with a larger average grain size for Cat.B. Although Cat.A and Cat.C have a large difference in Pt loading, their diffraction peak intensity is similar. This result presents a definite fact that there is no relation between the Pt loading and Pt grain size, further suggesting that a greater Pt loading is not always better.

**Fig. 4:**
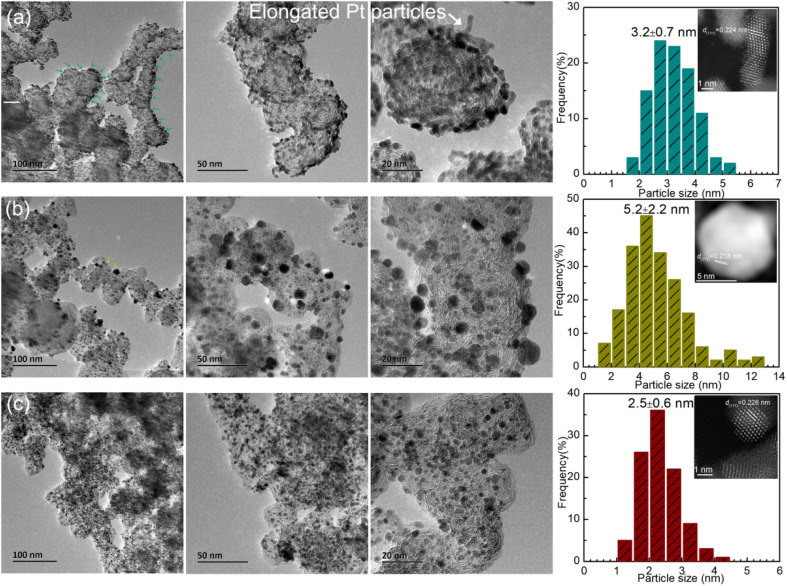
TEM images and particle size distribution of (a) Cat.A, (b) Cat.B and (c) Cat.C, where the insets show the Pt NP microstructure in terms of STEM-HADDF mode.

To better identify the morphology of the carbon support, Pt dispersion and microstructure, TEM and STEM-HAADF were performed, as shown in Fig. 4. It can clearly be seen that the carbon nanocrystal particles are diverse for the three catalysts. For Cat.A and Cat.B, the dimensions of the carbon nanocrystal particles range mainly from 40 nm to 60 nm. In contrast, the carbon nanocrystal dimension distribution of Cat.C is predominantly located between 20 and 30 nm with good graphitization. Many highly active catalysts utilize this type of carbon support to provide more dispersion sites for Pt NPs with high electrical conductivity and good corrosion resistance. Alternatively, the TEM images show that the Pt NPs are enriched at or near the support surface for Cat.A with an average size of approximately 3.2 nm (see Fig. 4a), and the elongated morphology of the Pt NPs (see the inset of Fig. 4a) differs from that of the high-performance catalyst reported in previous works.^27,33,34^ By contrast, for Cat.B (Fig. 4b), the main issue is the large Pt grain size (∼5.2 nm) rather than the Pt dispersion, which led to the exposure of less metal sites. However, it is apparent that there is a uniform dispersion of Pt NPs, and statistical analysis according to the TEM images in Fig. 4c gives the mean Pt particle size of ∼2.5 nm. This type of Pt/C catalyst is available from several well-known catalyst manufacturers. It should be noted that the Pt grain size calculated according to the Scherrer formula in the above-mentioned XRD analysis is consistent with that by microscopic statistics for Cat.C, suggesting the good Pt dispersion and particle size uniformity in Cat.C. The deviation in Cat.A and Cat.B by the two methods is ascribed to their inferior Pt dispersion, Pt aggregation and large Pt particle size distribution. Therefore, considering the Pt active component, Cat.C will have highly efficient activity due to its uniform size and high dispersion, which are indispensable for excellent activity and durability.

**Fig. 5:**
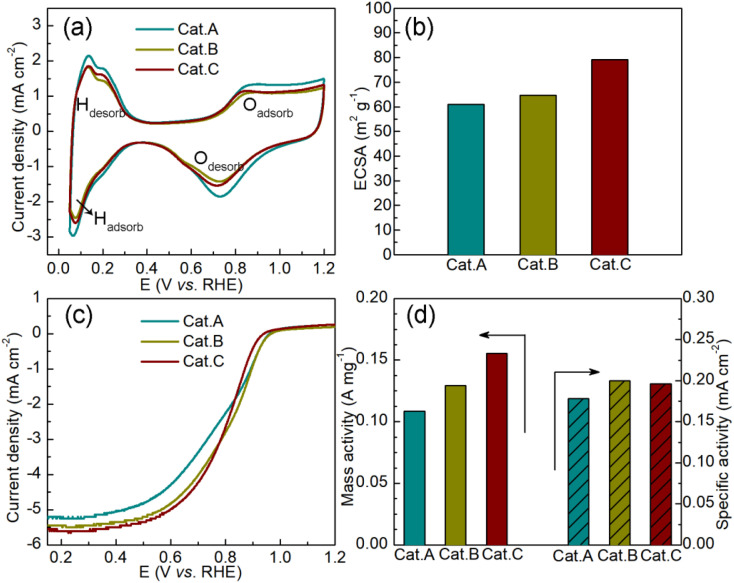
(a) CV curves of the three commercial Pt/C catalysts and (b) corresponding ECSA, (c) LCV curves of the ORR reaction in 0.1 mol L^−1^ HClO_4_ and (d) the corresponding mass activities and specific activities.

Subsequently, the cyclic voltammetry (CV) curves were recorded in N_2_-saturated acidic medium (0.1 mol L^−1^ HClO_4_) to assess the ORR activity of the three commercial catalysts. In Fig. 5a, two typical responses can be observed, which are related to hydrogen adsorption/desorption and Pt/Pt–O oxidation/reduction. Based on the CV curves, we calculated the electrochemically active surface area (ECSA), as shown in Fig. 5b. The improved ECSA of Cat.C provides evidence for the uniform dispersion of Pt NPs on the graphitized carbon support, suggesting that Cat.C is more electrochemically accessible compared to Cat.A and Cat.B. This feature plays a crucial part in enhancing the electrocatalytic kinetics of the ORR.^2^Fig. 5c shows the LCV curves of the three catalysts. Obviously, Cat.C exhibited the highest limiting current densities at a rotation speed of 1600 rpm. Alternatively, it should be noted that Cat.B has the highest half-wave potential (*E*_1/2_) of 0.810 V, followed by Cat.C and Cat.A with values of 0.799 V and 0.783 V, respectively, and the kinetic current density (*J*_*k*_) has a similar trend to *E*_1/2_ (*J*_*k*(Cat.B)_: 5.1908 mA cm^−2^ > *J*_*k*(Cat.C)_: 5.0965 mA cm^−2^ > *J*_*k*(Cat.A)_: 4.6229 mA cm^−2^). Based on this, the mass activity (MA) and specific activity (SA) were deduced to better understand the intrinsic activity, as shown in Fig. 5d. In comparison with Cat.C, the lower MA of Cat.B with high surface area is probably due to the large Pt NP size and lower ECSA. The SA of Cat.C illustrates high intrinsic ORR activity due to its relatively high SA. These results demonstrate the favorable properties of Cat.C in enabling good ORR kinetics, which is in agreement with the prediction though the systematic characterization of the three commercial Pt/C catalysts.

### 3.2 Catalyst degradation analysis

**Fig. 6:**
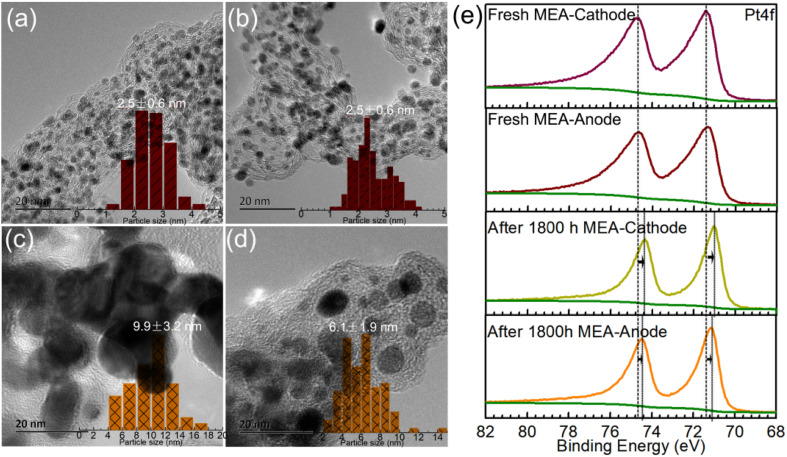
TEM micrographs of Pt component on as-made fresh MEA (cathode (a) and anode (b)) and after operating for 1800 h in MEA test in a stack of real fuel cell vehicle application (cathode (c) and anode (d)). The micrographs exhibit a significant discrepancy in the degree of Pt aggregation at the cathode and anode of the fuel cell. (e) Comparison of the high-resolution Pt 4f spectra of the Pt/C catalysts before and after operating for 1800 h.

The optimized Cat.C was translated to a real-world MEA to test its real vehicle performance by Hydrogen Energy Co., Ltd. The detailed operation information through testing cars in practice is presented in Section 2.3. The morphologies of the Pt/C catalysts on the as-made fresh MEA and MEA after operating for 1800 h in the real vehicle test were first investigated to compare their variations. As shown in Fig. 6, Pt/C catalyst degradation occurred with an apparent loss in Pt surface area after operating for 1800 h, which is associated with the crystal growth of platinum. The Pt NPs were uniformly dispersed on the 20 ∼ 30 nm carbon sphere support for both Pt/C catalysts of the as-made MEA cathode and anode (see Fig. 6a and b, respectively), where the particle size distribution of Pt/C by statistical analysis according to the TEM images is close to quasi-normal and predominantly located 2.5 nm. However, for the Pt/C catalysts after operating 1800 h, the particle size increased obviously to more than 4 nm, and sub-3 nm NPs were rarely observed (Fig. 6c and d). Upon closer inspection of Fig. 6c and d, it is evident that the Pt NPs in the cathode are significantly larger than that in the anode with time. The mean Pt particle size of the cathode catalysts was determined to be 9.9 ± 3.2 nm, which is nearly 4-fold that before use (2.5 ± 0.6 nm), greatly reducing the exposure of metal sites. Alternatively, the Pt particle size of the anode catalyst after 1800 h was relatively smaller compared to that of the degraded cathode catalysts, but still larger than that before use. Moreover, the XPS spectra also support Pt coalescence during fuel cell operation. As shown in Fig. 6e, the Pt 4f peaks of the Pt/C catalysts after operating for 1800 h in real automotive application shifted to lower BEs compared to that of the catalysts before use, indicating the coalescence of the Pt NPs because Pt coalescence reduces the exposed surface of Pt NPs and further decreases the surface oxide of Pt. Also, it is noteworthy that the shift value of the cathode Pt 4f is larger than that of the anode after running for 1800 h. This phenomenon can be ascribed to the violent three-phase interfacial reaction (ORR) occurring at the cathode side. Overall, the morphological changes in the facets of the Pt NPs during fuel cell operation make them easily deactivated, resulting in a degradation in their catalytic activity.

**Fig. 7:**
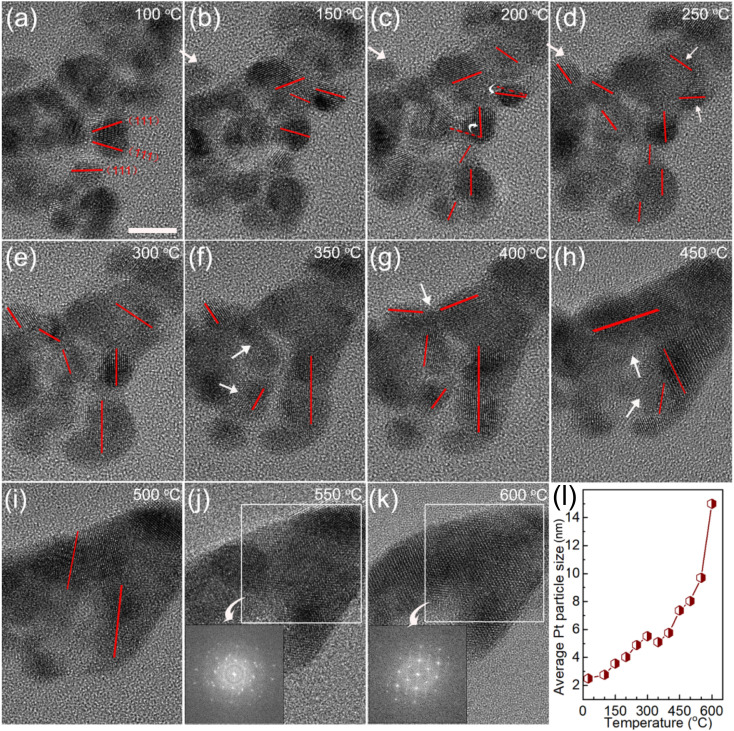
(a–k) *In situ* TEM images of Cat.C during the heat treatment process and schematic representation of the Pt particle-size-dependent merging with an increase in temperature. Scale bar, 5 nm. (l) Relationship of average Pt particle size and temperature.

To make the growth processes of the Pt NPs clear, *in situ* TEM study of Cat.C driven by temperature was explored to directly observe the evolution of the Pt NPs, as displayed in Fig. 7. It can be seen that at low temperature, the Pt particles with the adjacent orientation of (111) facets are prone to coalesce (see Fig. 7a). With an increase in temperature, the individual Pt particles got closer and their orientation started to rotate, until their (111) facets were aligned. Once the crystal facet matching is established, coalesce will occur (see Fig. 7b–i), and eventually a large particle is formed (see Fig. 7j and k). This phenomenon is similar to the growth mechanism of Au NPs with surface ligands.^35^ Accordingly, the first description of the growth of the Pt NPs in the discussion is confirmed, that is, the Pt NPs migrate with oriented rotation on the carbon support surface, and then coalesce, which leads to a significant reduction in the number of active sites. Alternatively, Fig. 7m shows the relationship between average Pt particle size and temperature, indicating that with an increase in temperature, the average size of the Pt NPs gradually increases until the coalescing is complete.

**Fig. 8:**
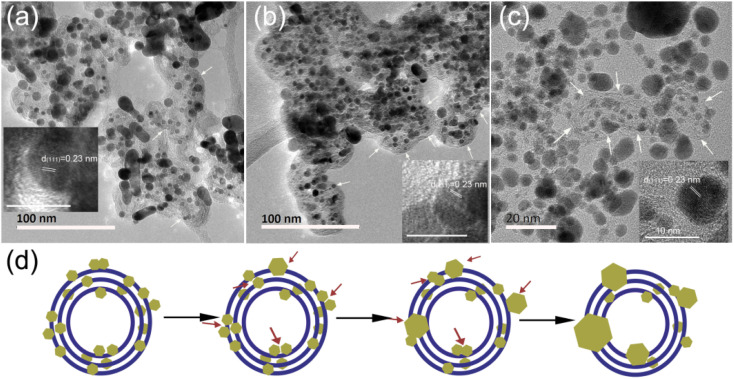
TEM images of the Pt/C catalyst after 1800 h practical application (cathode (a) and anode (b)), and after heating at 600 °C in the *in situ* DENSsolutions climate air holder (c). By comparing the changes in the Pt/C catalysts under the two conditions, a schematic illustration of Pt particle growth (d).

Moreover, we compared the variations in the Pt/C catalysts in two cases, *i.e.*, operated for 1800 h in the real vehicle application and the fresh catalyst heated in the *in situ* TEM sample rod. As shown in Fig. 8a–c, Pt particle coalescence easily occurred at or near the carbon support surface, and the Pt particles inside the carbon support are relatively stable in both cases, as summarized schematically in Fig. 8d. This similar observation of grown Pt particle verifies that the final apparent state of the Pt particles is consistent whether under complex REDOX reaction conditions or a single heating condition. Here, in our observation, the process of small Pt NPs dissolving and redepositing on the carbon support did not exist under a single temperature drive. The rotational orientation, migration and merging of Pt particles at or near the support surface are the primary reason for the catalyst deactivation. Thus, the on-demand control of the Pt particle-support interactions is crucial for electrocatalyst activity and stability improvement, which is an effective strategy to alleviate the deactivation of the catalyst.

## 4 Conclusion

In summary, the merits and disadvantages of commercial Pt/C electrocatalysts were identified by investigating their support and Pt active component. The superior carbon support of commercial Pt/C catalysts was characterized to have a fairly good pore structure and high graphitization degree. Cat.C with a superior carbon support has 2.0 ∼ 3.0 nm Pt NPs, indicating the presence of more active sites, and thereby has an excellent ORR performance (ECSA: 79.2 m^2^ g^−1^, MA: 0.1555 A mg^−1^, and SA: 0.2001 mA cm^−2^). Then, Cat.C was assembled into an MEA for real automotive application for 1800 h. By comparing the variations in the catalysts before and after application, the reason for their deactivation was proposed. The coalescence of Pt NPs during operating is considered to be the main reason for the weakened electrocatalytic ORR. Moreover, by studying the Pt particle-size dependent variations *in situ* using TEM, the trajectories of oriented attachment were disclosed by observing the coalescence events of the Pt NPs. These results provide specific guidelines toward the choice of higher-performing commercial Pt/C catalysts and carbon support optimization for the future development of higher-performing electrocatalysts.

## Author contributions

W. H. He and G. T. Xu designed the work. W. H. He, Y. J. Xiang and M. D. Xin performed the experiments and analyzed the data. L. M. Qiu, W. Y. Dong and W. H. Zhao participated in the data analysis and manuscript write. Y. X. Diao and A. G. Zheng participated in the modification of the manuscript. All the authors have given approval to the final version of this manuscript.

## Conflicts of interest

There are no conflicts to declare.

## Supplementary Material
